# Reading and writing from right to left after anterior cerebral artery stroke

**DOI:** 10.1590/1980-5764-DN-2023-0044

**Published:** 2024-04-15

**Authors:** Lílian Reuter, Guilherme Carvalho, Alex Reuter, Paula Caldeira

**Affiliations:** 1 Rede Sarah de Hospitais de Reabilitação, Belo Horizonte, MG, Brazil.

**Keywords:** Dyslexia, Agraphia, Stroke, Infarction, Anterior Cerebral Artery, Rehabilitation of Speech and Language Disorders, Motor Cortex, Dislexia, Agrafia, Acidente Vascular Cerebral, Infarto da Artéria Cerebral Anterior, Reabilitação dos Transtornos da Fala e da Linguagem, Córtex Motor

## Abstract

This is the case report of a woman who started to write and read from right to left after anterior cerebral artery stroke, affecting the left supplementary motor area. No cases were found in the literature with exactly the same characteristics. She has been able to read and write faster after rehabilitation approach at Sarah Network of Rehabilitation Hospitals, in the Belo Horizonte city unit, Brazil, despite the maintenance of the inversion. She returned to her previous activities in an adaptive way. It was discussed how the dysfunction in this cerebral area and its connections may disturb the reading strategy and direction.

## RESUMO.

Relato do caso de uma mulher que passou a escrever e ler da direita para a esquerda após um acidente vascular encefálico isquêmico de artéria cerebral anterior, acometendo área motora suplementar esquerda. Não foram encontrados casos na literatura exatamente com as mesmas características. Durante a participação da paciente no programa de reabilitação neurológica da Rede Sarah de Hospitais de Reabilitação, unidade Belo Horizonte, foram observados ganhos na agilidade de leitura e escrita, ainda que mantendo a inversão, e retorno às suas atividades de forma adaptada. Realizou-se discussão de como o comprometimento dessa área e de suas conexões pode perturbar a estratégia de leitura e sua direção.

## INTRODUCTION

The reading and writing processes are recent cultural events^1–3^. The human genome has not had time to modify itself and develop brain circuits suitable for reading. Hence brain circuits related to other cognitive tasks take part in this process. Learning to read requires the development of efficient links between language and vision areas^1,2,4^. Some kinds of brain injuries contribute elucidating neural circuits related to visual letter string processing^5^. They also support an understanding of how this information is distributed to the different regions primarily intended for spoken language. The actual brain connectivity is probably much more plentiful than we know so far^1–3^.

The present article aimed to report the case of a woman who started writing from right to left after a stroke in the anterior cerebral artery (ACA) branches related to medial surface of left hemisphere, affecting frontal and parietal areas, including great extension of supplementary motor area. In a recent review, Hertrich et al. (2016) pointed out that this is a neglected region in brain language processing models, despite numerous evidence in this regard^6^.

Several neurological impairments can follow ACA territory infarction, including weakness, sensory loss, apraxia, callosal disconnection sign, akinetic mutism and motor neglect, language and cognitive deficits, as well as urinary incontinence. ACA infarcts represent around 0.3 to 6% of acute ischemic strokes.^7^

Although there are several descriptions of acquired visuospatial processing disturbances affecting reading and writing strategies, we found similar but not identical cases as related in the present article^8^.

There is a case of a patient who acquired mirror reading and writing after traumatically brain injury. Besides reading and writing from right to left, each character was written in backwards^1,9^.

Balfour et al. carried out a control-case study. They identified some kind of mirrored writing in 15 of 86 patients (17.5%). There were different patterns of impairment, like a mirrored signature or inversion of some letters. They highlighted the importance of evaluating writing with the non-dominant hand, where generally the disorder usually arises. They pointed out that more cases could be seen with this procedure in clinical settings^10^.

## METHODS

### Context

This case study was approved by the Ethics Committee of the Associação das Pioneiras Sociais, CAAE 65529522.2.0000.0022 of the Sarah Network of Rehabilitation Hospitals. The patient consented to the use and disclosure of images and personal information, preserving her identity, signing the Informed Consent. She was followed between July 2019 and December 2020.

### Case history

**Figure 1:**
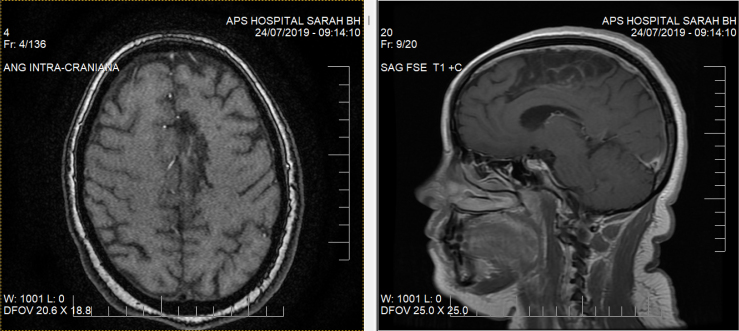
Magnetic resonance imaging showing encephalomalacia area in branches of the left anterior cerebral territory, covering most of supplementary motor area (Brodmann area 6).

A 36-year-old, previously healthy woman, kindergarten teacher, presented with stroke in December 2018. Magnetic resonance imaging showed an extensive cortical and subcortical parasagittal frontoparietal lesion in the left ACA area, affecting a large part of the supplementary motor area (Figure 1).

She was admitted to the Sarah Network of Rehabilitation Hospitals, in the Belo Horizonte city unit, Brazil, in July 2019, accompanied by her brother. The main deficits were predominantly right crural hemiparesis, and mild cognitive and language impairment. Her main expectation was “to walk better”. With more targeted questions, the brother reported that he observed slowness in reasoning.

A neuropsychological assessment was carried out to better characterize cognitive deficits. The semantic fluency test (animals)^11^, trail-making test (TMT)^12^, and Rey auditory-verbal learning test (RAVLT)^12–14^ were administered. Furthermore, an informal praxis examination was undertaken. Table 1 shows some information on the assessment date as well as a comparison of the fluency test and RAVLT results.

**Table 1:** Neuropsychological assessment results.

Dates	Tests*	Rey auditory-verbal learning test10 (RAVLT)								Fluency^11^	
		A1*	A2	A3	A4	A5	B	A6	A7	Animals	F^12^
27/08/19		4 (-1.5)	3 (-3.5)	5 (-2.0)	5 (-3.7)	7 (-4.0)	NA†	NA	5 (-3.6)	10 (-1.2)	6 (-1.9)
28/11/19		4 (-1.5)	7 (-1.1)	9 (-0.8)	9 (-1.6)	11 (-1.3)	1 (-4.1)	6 (-3.1)	6 (-2.5)	14 (-0.6)	11 (-0.9)

Source: authors.

* Notes: In parentheses the Z-scores of the tests RAVLT^10^, fluency animals^11^ and fluency F^12^. Used the total sample of RAVLT and the group of 11 years without animal fluency;

† NA: not applied, due to difficulty in learning list A in the first application. The memory A7 was done after 20 minutes, with two other tests as interfering activities (countdown from 50 to 30, and tower of London).

Deficit in verbal learning and memory was identified in addition to slowness in fluency and TMT tests. In TMT, we did not consider the time, given the increased time to execute part A, which requires the linking of numbers. The time to make gestures was increased too.

The patient was followed up between July 2019 and December 2020.

#### First stage: cognitive rehabilitation

A first intervention plan was proposed, with the emphasis on gaining agility to perform cognitive activities. She participated in group activities, in which board games were used as cognitive stimulation. It was also carried out a computerized cognitive training program targeted at stimulating attention, memory, calculation, and planning skills. These activities were part of the rehabilitation program as a whole, which aimed to improve activities of daily living and locomotion. This stage lasted between August and November 2019.

The patient, her brother, and the team observed an improvement in social participation. She was able to express her ideas easily in addition to reasoning with greater agility.

In the comparative evaluation (Table 1), there was evidence of improvement in the results of verbal learning and fluency tests. However, some deficits remained. The slowness to execute the TMT was highlighted. Due to the struggle to recognize the letters in part B of this test, a more detailed language assessment was performed.

#### Second stage: writing and reading disfunctions approach

The Montreal-Toulouse Language Assessment Battery — Brazilian Version (MTL-Brasil)^15^ was administered. She presented the characteristics of anomic aphasia: coherent but slow speech, decrease in verbal fluency, and use of short sentences, with pauses and hesitations. She used to write inside her mind during anomic episodes to help her lexical access. She had struggles understanding abstract content phrases and making inferences. Table 2 shows the most relevant results of MTL.

**Table 2:** Montreal-Tolouse Battery results (MTL-Brasil).

Tasks	Admission	End
	Time in seconds	
Most frequent word “cruz”	4	2
Least frequent word “tórax”	17	15
No-word “duta”	16	14
Usual word writing “macaco”	11	6
Written under sentence dictation “Os vidros do quarto não têm sido bem limpos”	142	114
	Number of words	
Written narrative speech test	12	17
	Correct items	
Written comprehension of the text	6/9	9/9
Semantic fluency animals: in a minute and a half	7	12
Phonemic fluency: words starting with “m” in one and a half minute	8	7

Source: authors.

Notes: *Cruz:* cross; *Tórax*: thorax; *Macaco*: monkey; *Duta*: this is a pseudoword; *Os vidros do quarto não têm sido bem limpos*: The windows in the room have not been thoroughly cleaned.

At that time, the difficulty in reading and writing was evident. She read from right to left, and this was possible only if words were spelled in capital letters. As it was a laborious, slow reading, she was able to interpret only simple words and phrases.

**Figure 2:**
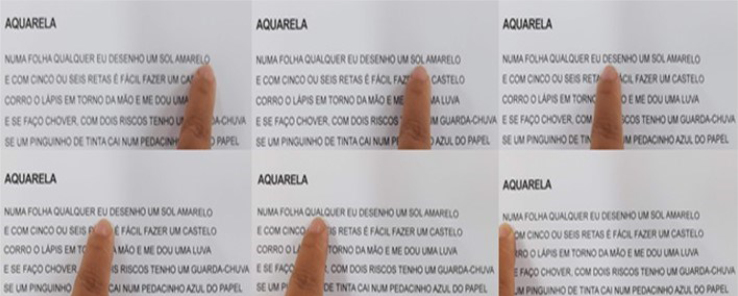
Reading from right to left, letter by letter, the lyrics of a popular Brazilian song. Photo with the patient pointing.

The patient moved her index finger from right to left across each letter, starting decoding at the end of the sentence. Beginning from the right, she had to pass through each letter, performing the decoding mentally. Afterward, the sentence was read aloud. Sometimes, it was necessary to read the sentence again in order to memorize it all (Figure 2). She could not read cursive.

**Figure 3:**
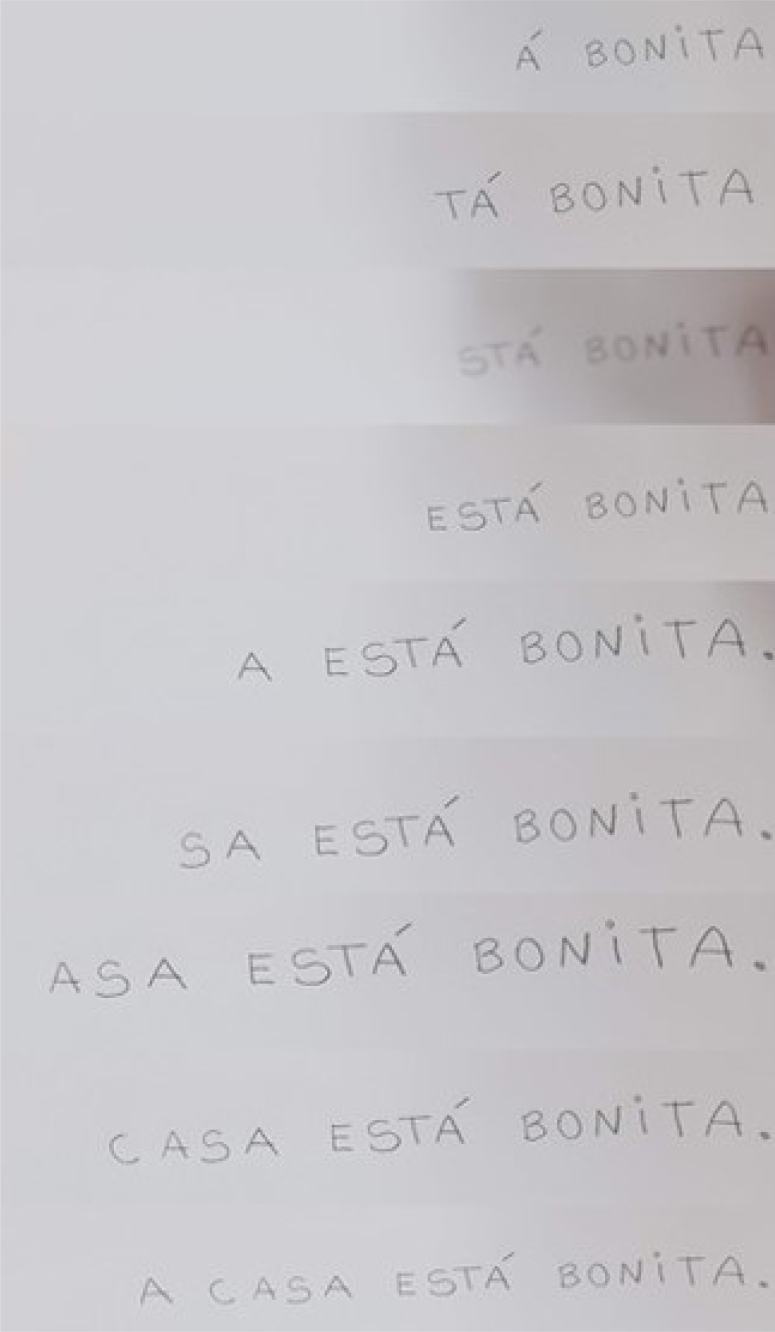
Writing from right to left.

The writing followed the same standard, from right to left. She used to measure the enough sheet space to write the entire phrase and “conclude” in the left margin. Each word was spelled letter by letter, from the last to the first. As the writing in cursive was also not possible, it was done in capital letters. When a word was finished, the next one was also initiated from the last letter, respecting the spacing between words (Figure 3).

In the beginning, the writing presented a decrease in speed and micrography. She kept writing with her dominant hand. The graphomotor pattern became normal with a cylindrical rubber pencil grip 2 cm in diameter. She started writing with capital letters in a regular size after this support.

In order to better characterize spatial dysfunction, the odd-one-out test was performed^1,16^. This task is presented in five rows with three stimuli for each one. There are rabbit drawings in the three first lines and the word “rabbit” in the other two. On each line, one of the three stimuli is the “odd-one-out”. In the first row, the different drawing has his head to the left, while the other two are to the right. In the second, the “odd-one-out” is upside down. In the third, they are in the same position, but a rabbit has four ears. On the fourth line, the different word is written backwards (“OHLEOC”). On the fifth one, the word is written mirrored.

Among the drawing rows, she struggled to recognize the different drawing on the first row, where the “odd-one-out” was mirrored. She did not get to perceive the right-facing rabbit among the other left-facing rabbits. The word rabbit written mirrored was pointed with a feeling of satisfaction. She said: “I read in this way”.

After evaluating the linguistic functions, a second stage of intervention was proposed, focusing on writing exercises such as copying, categorization, completion of sentences with options, identification of words with wrong spelling vs. correct spelling, and spontaneous writing of words with complex syllables. In the last task, there was the support of movable letters, subsequently, spontaneous writing of sentences, and interpretation of abstract passages.

She had weekly rehabilitation sessions, both at home and in the hospital outclinic. The patient showed satisfactory involvement in the tasks, having strong family support during the process. This second stage took place between January and October 2020.

In the assessment after the intervention period, the mirrored reading was still present, but she was able to mentally reverse the order of words more quickly before saying them.

There are more details of the MTL-Brazil results in Table 2. It is possible to observe a reduction in the time spent on most of the tasks. The shorter time for high-frequency words remained, as well as the longer time for irregular words, and longer latency for pseudowords (lexicality effect). Despite the gain in reading agility, it remained laborious and with greater support from the phonological route instead of lexical^17^.

The reading was faster and more precise when the words were presented in a mirror (similar time of a child in literacy phase — Table 3). This task was performed after the rehabilitation program, without previous data.

**Table 3:** Mirror reading.

Mirror reading	Time in seconds
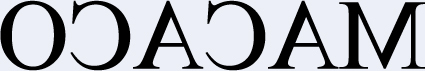	1 second
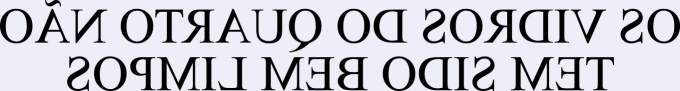	7 seconds

Source: authors.

After interventions, she was able to produce larger texts and interpret small texts with abstract content. Verbal expression and comprehension were also improved, with a reduction in anomie episodes, enhanced verbal fluency, and understanding of long sentences. Despite not presenting a greater number of words during the oral speech test, she started to improve her communicative initiative and was more confident. There was a decrease in episodes of hesitation during speech as well.

The improvements contributed to psychological and social repercussions. She became more confident and active and returned to work at the family's school with reduced hours. The activities were adjusted for her current conditions, doing administrative tasks. Because of the disruption of school activities due to the Covid pandemic, she started to produce popsicles-like, put in bags. This production required the writing of labels and menus, besides sales organization.

## DISCUSSION

The supplementary motor area is in the frontal region, in the medial part of Brodmann area 6. It participates in the processing of various cognitive and language functions, working as a convergence zone^6^. There is greater recruitment of the left supplementary motor area during literacy training for children and adults^4,18,19^.

The patient's way of reading and the effort in the reading process suggests a dysfunction in the reading circuit responsible for the visual recognition of the word, access to the lexicon, and its meaning^4^. The parietal and frontal cortices transmit outputs toward the visual areas in order to select the region of space from which the reading begins. Injuries in these areas can disrupt reading strategy and direction^1^.

Another theory that supports the case is the “mirror theory”, which states that each learning of an image in one hemisphere is accompanied by learning in a mirror in the other hemisphere. The “symmetrization” transfers memory objects between the hemispheres through the corpus callosum. During the evolutionary process, the individual whose visual system was able to generalize in a mirror was favored. This factor facilitates quick object and natural world recognition. So, learning to recognize an image leads to immediate recognition of the mirror's symmetrical shape^1^.

The visual system architecture is prone to reading. Nevertheless, there is more effort to visual recognition of mirrored letters. This might, for instance, contribute to the confusion between the letters “q” and “p”. During the literacy process, children write in a mirror until they learn to disregard the mirror image of the letters, from the moment they become fluent readers. They start using the left hemisphere for reading and neglect the other^1^.

In the present study, the symmetry alteration was not only restricted to reading, as demonstrated in the odd-one-out test. However, it brought greater impact on this activity.

Despite being approached in a chronic stage of brain injury, it was possible to verify gains in reading and writing, such as speed, fluency, and readability. Although the visuospatial problems continued, the patient developed the ability to mentally invert words, favoring a greater agility in reading and writing practices.

As a case report related to clinical follow-up, some important procedures could have been implemented in order to favor the disorder comprehension and interventions. Considering the visuospatial deficit hypothesis, a more comprehensive assessment of this cognitive area could have been performed. At some point in the rehabilitation process, linguistic problems received more attention.

Balfour et al. discussed the importance of evaluating writing with the non-dominant hand, a situation in which the manifestation of mirrored writing is much more frequent^10^. As the patient was able to write with her dominant hand during the rehabilitation process, this was not a concern at that moment. However, even without this data, it was possible to observe spatial disorder in the reading, the writing of the dominant hand, and the odd-one-out test.

In conclusion, brain lesions favor the discovery of the systems that the competent reader uses to read and write. In the rehabilitation process, it is necessary to deeply know the individual, their current limitations and potential, so that the professional can act with clarity and specificity.

In this process, the rehabilitation team improves the knowledge about the patients’ needs and motivation. It is possible to assist them in the development of their global potential, transforming individuals back into agents of their existence and able to choose the paths to follow, albeit in an adapted way.
